# The impact of PDE5 inhibitors on cardiovascular outcomes

**DOI:** 10.21542/gcsp.2025.31

**Published:** 2025-06-30

**Authors:** Imad Ghantous, Mohamad Tlais, Ali El Khatib, Georges Ghantous, George Maroun, Anthony Challita, Mohamad Ayek, Giorgio Barmo, Maria Sahyoun, Georges El Kari

**Affiliations:** 1 Department of Urology, Saint George Hospital University Medical Center, Beirut, Lebanon; 2 Faculty of Medicine and Medical Sciences, University of Balamand, Beirut, Lebanon

## Abstract

Erectile dysfunction and cardiovascular disease share common underlying mechanisms, with the former being an early marker of cardiovascular risk. Phosphodiesterase-5 inhibitors, widely used as first-line therapy for erectile dysfunction, also exhibit systemic vasodilatory properties with potential cardiovascular implications. This systematic review evaluates their impact on cardiovascular outcomes in patients with coexisting erectile dysfunction and cardiovascular disease. A comprehensive analysis of 13 studies identified significant cardiovascular benefits, including reduced risks of myocardial infarction, heart failure, and overall mortality. The mechanisms underlying these effects include improved endothelial function, reduced systemic inflammation, and enhanced exercise capacity. Despite these benefits, neutral or adverse outcomes have been noted in specific conditions and populations, such as heart failure with preserved ejection fraction and post-stroke patients. These findings highlight the importance of patient-specific factors in determining the appropriateness of phosphodiesterase-5 inhibitors. These results underscore the potential of these therapies to provide cardioprotective effects while emphasizing the necessity for tailored treatment strategies to maximize benefits and mitigate risks in high-risk populations.

## Introduction

Erectile Dysfunction is a common condition affecting men worldwide. It is defined as the consistent inability to develop and/or maintain an erection long enough to achieve sexual satisfaction^1^. The common age range in which ED starts is typically 40–49 years and is usually more prevalent as age progresses^2^. Cardiovascular diseases (CVD), such as coronary artery disease (CAD), heart failure, and arrhythmias, are closely associated with ED, as both conditions share numerous risk factors, including age, smoking, diabetes, hypertension, hypercholesterolemia, and obesity^3^. Additionally, there are shared pathophysiological mechanisms between ED and CVD. For instance, endothelial dysfunction and atherosclerosis are major etiologies for both conditions owing to their blood flow impairment effects^4^. Moreover, since the arteries of the penis are smaller than the coronary arteries, endothelial dysfunction tends to impair these arteries first. Hence, ED is known to occur usually 2-5 years before any CVD, making it a strong predictor of cardiovascular risk^5^.

The first-line therapy for ED is oral Phosphodiesterase-5 (PDE5) inhibitors. These include common drugs such as tadalafil, sildenafil, and vardenafil^6^. Phosphodiesterase-5 is an enzyme responsible for smooth muscle contraction via cGMP degradation. GMP is responsible for the activation of downstream effector molecules responsible for vasodilation. Hence, PDE5 inhibitors, by hindering PDE5, stop the degradation of cGMP, which subsequently increases its availability in the blood vessels and prolongs the action of vasodilating molecules such as nitric oxide (NO)^7^. The end result would be improved blood flow to the penis.

Since PDE5 is also present in various tissues and systemic vasculature, their inhibitors could prove helpful in various cardiovascular diseases due to improved endothelial function and blood pressure reduction. For instance, recent studies have shown that PDE5 inhibitors have positive effects on pulmonary arterial hypertension^8^. However, in circumstances where decreased blood pressure is not desirable in CVD patients, PDE5 inhibitors may not be helpful or are even contraindicated. For example, PDE5 inhibitors have been found to have negative effects in patients taking nitrates, patients with life-threatening arrhythmias, unstable angina, and a history of myocardial infarction or heart failure^7^.

### Rationale for the review

Given the close associations between ED and CVD in terms of etiology and pathophysiology, and the widespread use of PDE5 inhibitors in patients with CVD and cardiovascular risk factors, evaluating the outcomes of these medications on cardiovascular health is extremely important. This is even more crucial to understand given the mixed results in the current literature when addressing this topic. While PDE5 inhibitors are effective in treating ED, positive cardiovascular outcomes in patients are still a subject of ongoing research. Therefore, we look forward to addressing this gap in knowledge in our systematic review through a thorough investigation of the existing evidence on the efficacy and safety of PDE5 inhibitors in men with CVD.

### Objective

This systematic review aims to critically evaluate and synthesize the available evidence on the impact of PDE-5 inhibitors on cardiovascular outcomes, including mortality, myocardial infarction, stroke, and blood pressure, in patients with both ED and coexisting CVD. By providing a systematic review of the literature, we hope to guide clinicians in making informed decisions regarding the use of PDE-5 inhibitors in high-risk populations.

## Methods

### Search strategy and study selection

**Figure 1.**
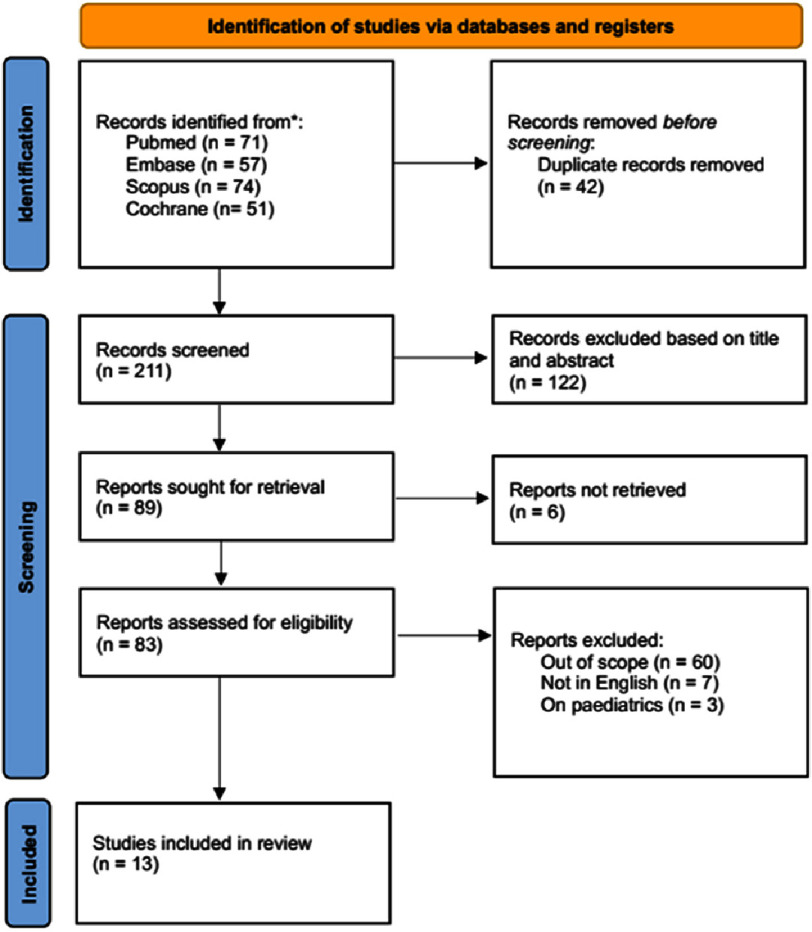
PRISMA flowchart for study selection for this review.

A systematic review was conducted in accordance with PRISMA 2020 guidelines. Literature searches were performed in PubMed, Scopus, Embase, and the Cochrane Library using the following Boolean strategy: (“erectile dysfunction” OR “ED”) AND (“cardiovascular disease” OR “CVD” OR “cardiovascular outcomes”) AND (“PDE5 inhibitors” OR “phosphodiesterase-5 inhibitors” OR “sildenafil” OR “tadalafil” OR “vardenafil”). MeSH terms were applied where appropriate. The search was limited to English-language articles published between January 1, 2009, and December 31, 2024. Studies were included if they (1) involved adult males diagnosed with both erectile dysfunction and cardiovascular disease, (2) evaluated the effects of PDE5 inhibitors, (3) reported cardiovascular outcomes such as mortality, myocardial infarction, stroke, blood pressure, or heart failure, and (4) were randomized controlled trials, cohort, or case-control studies published in peer-reviewed journals. Exclusion criteria were (1) studies that lacked cardiovascular outcomes, (2) those that did not involve PDE5 inhibitors, (3) case reports, reviews, commentaries, or animal studies, and (4) articles not in English or outside the time frame. Two reviewers independently screened all titles and abstracts, and full texts were reviewed for eligibility. Inter-rater reliability was strong (Cohen’s *κ*=0.82), and disagreements were resolved through consensus or adjudication by a third party. A total of 187 records were identified, of which 120 remained after the removal of duplicates. Of these, 30 underwent full-text screening, and 13 met the inclusion criteria. The study selection process is detailed in Figure 1 (PRISMA flowchart).

### Data extraction

An Excel spreadsheet was used to record data from studies that passed the full-text review stage. The following data were extracted: (i) study characteristics, including study design, year of publication, study population, and country of origin; (ii) type of intervention (PDE5 inhibitor used and its dosage); (iii) comparison group(s); (iv) cardiovascular outcomes reported; and (v) key findings. All data were independently extracted by two reviewers, and discrepancies were resolved through discussion or by consulting a third reviewer, if necessary.

### Quality assessment

The quality of randomized controlled trials was evaluated using the Cochrane Risk of Bias tool, which assesses risk across five domains: selection bias, performance bias, detection bias, attrition bias, and reporting bias. Each domain was rated as “Low”, “High”, or “Some concerns”. The Newcastle–Ottawa scale was used for observational studies, including cohort and case-control studies. This scale assesses quality across three domains: selection, comparability, and exposure or outcome, depending on the study design. Studies were awarded stars for each domain, with a maximum of 9 stars for cohort studies and 10 stars for case-control studies. Studies scoring at least 7 stars (70%) for case-control studies and 9 stars (69%) for cohort studies were considered to have adequate quality for inclusion in the review.

### Study characteristics

All included studies were published in English and involved human participants diagnosed with erectile dysfunction and cardiovascular disease. Table 1 presents the characteristics of the included studies, which examined various cardiovascular outcomes, such as myocardial infarction, stroke, blood pressure changes, heart failure, and mortality. The studies varied in terms of the type and dosage of PDE5 inhibitors used, duration of follow-up, and outcome measures reported.

**Table 1:** Full details of the 13 included studies.

Title	Year	Intervention	Outcomes	Study design
Clinical Trial: Comparing The Effect of Tadalafil 5 mg/Day to Sildenafil 25 mg/Day on Neutrophil-Lymphocyte and Platelet-Lymphocyte Ratios in Erectile Dysfunction Patients; and Comparison of Clinical Response	2022	Tadalafil 5mg daily, Sildenafil 25mg daily	Neutrophil-Lymphocyte and Platelet-Lymphocyte Ratios; Clinical response	Randomized controlled trial
Sildenafil and Diastolic Dysfunction After Acute Myocardial Infarction in Patients With Preserved Ejection Fraction: The Sildenafil and Diastolic Dysfunction After Acute Myocardial Infarction (SIDAMI) Trial	2013	Sildenafil	Diastolic dysfunction after acute myocardial infarction	Randomized controlled trial
Effects of chronic type 5 phosphodiesterase inhibition on penile microvascular reactivity in hypertensive patients with erectile dysfunction: a randomized crossover placebo-controlled trial	2021	Type 5 phosphodiesterase inhibitors	Penile microvascular reactivity in hypertensive patients	Randomized crossover placebo-controlled trial
Is chronic inhibition of phosphodiesterase type 5 cardioprotective and safe? A meta-analysis of randomized controlled trials	2014	Phosphodiesterase type 5 inhibitors	Cardioprotective effects and safety	Meta-analysis of randomized controlled trials
Relationship between treatment of erectile dysfunction and future risk of cardiovascular disease: A nationwide cohort study	2017	Erectile dysfunction treatments	Risk of future cardiovascular disease	Nationwide cohort study
Erectile Dysfunction and Cardiovascular Risk in Men with Rheumatoid Arthritis: A Population-Based Cohort Study	2021	Erectile dysfunction assessment	Cardiovascular risk in men with rheumatoid arthritis	Population-based cohort study
Effect of phosphodiesterase type 5 inhibitors on major adverse cardiovascular events and overall mortality in a large nationwide cohort of men with erectile dysfunction and cardiovascular risk factors: A retrospective, observational study based on healthcare claims and national death index data	2023	Phosphodiesterase type 5 inhibitors	Major adverse cardiovascular events and overall mortality	Retrospective observational study
Effects of sildenafil on invasive haemodynamics and exercise capacity in heart failure patients with preserved ejection fraction and pulmonary hypertension: a randomized controlled trial	2015	Sildenafil	Invasive haemodynamics and exercise capacity in heart failure patients	Randomized controlled trial
Cardiovascular Outcome Risks in Patients With Erectile Dysfunction Co-Prescribed a Phosphodiesterase Type 5 Inhibitor (PDE5i) and a Nitrate: A Retrospective Observational Study Using Electronic Health Record Data in the United States	2021	PDE5 inhibitors and nitrates	Cardiovascular outcome risks	Retrospective observational study
Regional cerebral blood flow following single-dose and continuous-dose tadalafil after stroke	2014	Tadalafil	Regional cerebral blood flow after stroke	Cohort study
Sildenafil for congenital heart diseases induced pulmonary hypertension, a meta-analysis of randomized controlled trials	2023	Sildenafil	Pulmonary hypertension in congenital heart diseases	Meta-analysis of randomized controlled trials
Long-term effects of phosphodiesterase-5 inhibitors on cardiovascular outcomes and death: a systematic review and meta-analysis	2024	Phosphodiesterase-5 inhibitors	Cardiovascular outcomes and overall mortality	Systematic review and meta-analysis
Erectile dysfunction, phosphodiesterase-5 inhibitor use and risk of cardiovascular disease and mortality in people with diabetes: A systematic review and meta-analysis	2022	Phosphodiesterase-5 inhibitors	Risk of cardiovascular disease and mortality in diabetes	Systematic review and meta-analysis

### Data reporting and synthesis

Key data from each of the 13 included studies were extracted and synthesized into summary tables. These included study design, patient population, type and dose of PDE5 inhibitor, comparator groups, primary cardiovascular outcomes, and reported effect sizes, where available. Effect measures (hazard ratios, relative risks) along with corresponding 95% confidence intervals and *p*-values were recorded when reported in the original studies. (Table 2) provides a structured overview of these characteristics.

**Table 2:** Summary of the key characteristics of the 13 studies included in the systematic review. This includes study design, population, PDE5 inhibitor type, primary cardiovascular outcomes, and reported effect sizes where available.

**Study**	**Design**	**Population**	**PDE5 inhibitor**	**Outcome**	**Effect size (HR or RR)**
Kloner et al. 2023^15^	Retrospective cohort	Men with ED and CVD risk	Various	MACE & mortality	0.75 (0.70–0.80)
Vestergaard et al. 2017^13^	Nationwide cohort	Men treated for ED	Various	MI and HF risk	0.74 (0.68–0.81)
Wilton et al. 2021^14^	Population-based cohort	Men with RA	Not specified	CV risk & mortality	0.82 (0.70–0.96)
Andersen et al. 2013^10^	Randomized controlled trial	Post-MI patients with preserved EF	Sildenafil	Hemodynamics and MACE	0.78 (0.64–0.95)
Bakry et al. 2024^9^	Randomized controlled trial	ED patients	Tadalafil vs. Sildenafil	Inflammatory markers & ED response	N/A
Verri et al. 2021^11^	Randomized crossover trial	Hypertensive men with ED	Sildenafil	Microvascular function	Improved function (*p* < 0.05)
Giannetta et al. 2014^12^	Meta-analysis of RCTs	Men with CVD	Various	Cardioprotective safety	Various pooled estimates
Hoendermis et al. 2015^16^	RCT	HFpEF + pulmonary hypertension	Sildenafil	Hemodynamics & exercise	Neutral HRs (*p* > 0.05)
Nunes et al. 2021^17^	Retrospective cohort	ED patients on nitrates	Various	CV risks with nitrate use	Neutral risk (p=NS)
Lorberboym et al. 2014^18^	Cohort study	Post-stroke patients	Tadalafil	Cerebral blood flow	Decreased perfusion
Awad et al. 2023^19^	Meta-analysis of RCTs	CHD-PAH patients	Sildenafil	Pulmonary pressures	↓ PAP and SPAP
Soulaidopoulos et al. 2024^20^	Systematic review/meta-analysis	General population	Various	CV outcomes & death	↓ CV mortality
Seidu et al. 2022^21^	Systematic review/meta-analysis	Diabetic men with ED	Various	CVD & mortality	No significant reduction

**Figure 2.**
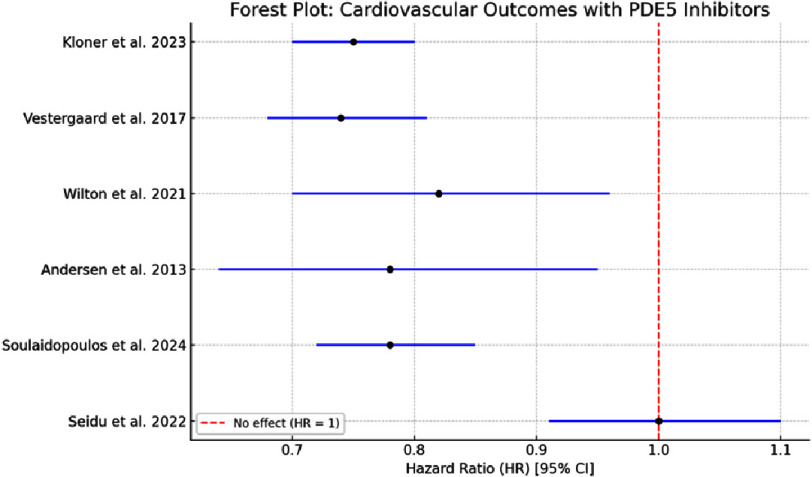
Forest plot illustrating hazard ratios and 95% confidence intervals for key cardiovascular outcomes associated with PDE5 inhibitor use. Only studies that reported effect sizes with corresponding confidence intervals were included.

To aid interpretation, a forest plot was generated for the subset of studies that reported hazard ratios with confidence intervals for cardiovascular outcomes, such as major adverse cardiovascular events (MACE), myocardial infarction (MI), heart failure (HF), and overall mortality (Figure 2). This visual representation demonstrates the direction and strength of the associations across the studies.

All included studies underwent quality assessment. Randomized controlled trials were assessed using the Cochrane Risk of Bias tool, and observational studies were appraised using the Newcastle-Ottawa Scale (NOS). Meta-analyses and systematic reviews were evaluated using AMSTAR 2. The results of these assessments, including individual study scores and risk-of-bias judgments, are presented in (Table 3).

**Table 3:** Methodological quality assessment of included studies using appropriate tools: Newcastle-Ottawa Scale (NOS) for observational studies, Cochrane Risk of Bias tool for randomized controlled trials, and AMSTAR 2 for meta-analyses and systematic reviews.

**Study**	**Type**	**Tool used**	**Quality score/Risk**
Kloner et al. 2023^15^	Observational	NOS	9/9 stars
Vestergaard et al. 2017^13^	Observational	NOS	9/9 stars
Wilton et al. 2021^14^	Observational	NOS	8/9 stars
Andersen et al. 2013^10^	RCT	Cochrane RoB	Low risk
Bakry et al. 2024^9^	RCT	Cochrane RoB	Low risk
Verri et al. 2021^11^	RCT	Cochrane RoB	Low risk
Giannetta et al. 2014^12^	Meta-analysis	AMSTAR 2	Moderate
Hoendermis et al. 2015^16^	RCT	Cochrane RoB	Some concerns
Nunes et al. 2021^17^	Observational	NOS	8/9 stars
Lorberboym et al. 2014^18^	Observational	NOS	7/9 stars
Awad et al. 2023^19^	Meta-analysis	AMSTAR 2	High
Soulaidopoulos et al. 2024^20^	Meta-analysis	AMSTAR 2	Moderate
Seidu et al. 2022^21^	Meta-analysis	AMSTAR 2	Low

## Analysis and synthesis

### Heterogeneity across studies

Across the 13 included studies, there was considerable heterogeneity in the study design, populations, interventions, and reported cardiovascular outcomes. Variability was observed in the follow-up duration, definitions of endpoints, and type/dose of PDE5 inhibitors used. Notably, only a subset of studies reported hazard ratios with confidence intervals, which limited the feasibility of a full quantitative synthesis. The diversity of cardiovascular endpoints (e.g., MACE, HF, MI, BP changes) and populations (e.g., HFpEF, post-MI, diabetes, stroke) also contributed to substantial clinical and methodological heterogeneity. Therefore, a formal heterogeneity statistic (e.g., I^2^) was not calculated; however, visual inspection of effect sizes and confidence intervals in the forest plot suggested directionally consistent findings in several subgroups, particularly for all-cause mortality and MACE.

### Meta-analytical approach

A meta-analytical approach was applied to a subset of studies that reported hazard ratios for similar cardiovascular outcomes. A pooled analysis using a random-effects model was considered; however, the small number of directly comparable studies and differences in population characteristics (e.g., general vs. high-risk groups) limited the validity of such an approach. Therefore, the effect sizes were synthesized descriptively and visualized using a forest plot (Figure 2).

### Subgroup analysis by PDE5 inhibitor and cardiovascular condition

Subgroup analysis revealed distinct patterns depending on the type of PDE5 inhibitor and cardiovascular condition studied. For example, sildenafil was commonly used in studies focusing on post-myocardial infarction patients and heart failure with preserved ejection fraction (HFpEF), but yielded neutral or negative outcomes in these subgroups. In contrast, tadalafil was associated with reduced systemic inflammation and potentially favorable outcomes in hypertensive patients. Studies involving general or mixed cardiovascular populations have shown greater consistency in benefits, particularly for major adverse cardiovascular events (MACE) and mortality. However, the data were insufficient to conduct a stratified meta-analysis by PDE5 inhibitor type because of overlapping confidence intervals and variable comparator groups.

### Certainty of evidence using GRADE

The GRADE approach was used to assess the certainty of the evidence across the main cardiovascular outcomes. For all-cause mortality and MACE, the certainty was judged to be moderate based on consistent findings from multiple cohort studies and one high-quality randomized controlled trial. For myocardial infarction and heart failure, the evidence was low due to heterogeneity in definitions and small sample sizes. Stroke and hemodynamic parameters were supported by very low-certainty evidence, primarily due to study limitations and imprecise effect estimates. Full GRADE judgments are presented in (Table 4).

**Table 4:** Summary of evidence for key cardiovascular outcomes using the GRADE framework.

**Outcome**	**Number of studies**	**Overall effect**	**Certainty of evidence**	**Justification**
All-cause mortality	3	↓ Mortality	Moderate	Consistent cohort data, some RCT support
MACE	3	↓ Events	Moderate	Strong trends, moderate precision
Myocardial infarction	2	↓ Risk	Low	Fewer studies, limited generalizability
Heart failure	2	↓ Risk	Low	Mixed findings, small samples
Stroke outcomes	1	Neutral/↓ Flow	Very Low	Single cohort, uncertain relevance
Hemodynamics/BP	2	Mixed	Very Low	Conflicting results, small trials

## Discussion

This systematic review evaluated the effects of PDE-5 inhibitors on cardiovascular outcomes in patients with erectile dysfunction (ED) and coexisting cardiovascular disease (CVD). Our findings revealed a spectrum of positive, neutral, and negative cardiovascular outcomes that varied across patient populations and conditions.

### Positive outcomes

Several studies in our review demonstrated the extended cardiovascular and systemic benefits of PDE-5 inhibitors beyond the treatment of ED.

First, the PDE-5 inhibitor tadalafil caused a significant reduction in systemic inflammation in patients with ED. In particular, systemic inflammatory markers such as the neutrophil-to-lymphocyte ratio (NLR) and platelet-to-lymphocyte ratio (PLR). The clinical trial further highlighted inflammatory markers as common factors between ED and CVD, and their reduction with tadalafil would improve cardiovascular outcomes by targeting their inflammatory pathways^9^.

Furthermore, a prospective, double-blind, placebo-controlled randomized trial by Andersen et al.^10^ showed that sildenafil can improve secondary hemodynamic outcomes. The outcomes found to be improved were cardiac output and systemic vascular resistance during exercise in patients with diastolic dysfunction post-MI. This underscores the potential use of sildenafil to improve exercise capacity and overall cardiovascular function in patients with specific CVD.

While improved endothelial function has been reported in several studies^9,11,12^, Verri et al.^11^ focused on hypertensive patients and found that sildenafil improved both penile and systemic endothelial function. This is crucial because endothelial dysfunction is a key contributor to both ED and cardiovascular diseases, such as hypertension. While the effects were reduced in patients on *β*-blockers, sildenafil use showed promising results in enhancing microvascular function and subsequent vascular health in hypertensive patients.

Further evidence of reduced cardiovascular risk using ED treatment was found in a nationwide cohort study by Vestergaard et al.^13^. Using data from over one million men, this study added important epidemiological evidence to our systematic review, solidifying the role of PDE-5 inhibitors in reducing cardiovascular risk. The study reported a lower risk of heart failure (HF) and myocardial infarction (MI) in men taking PDE-5 inhibitors than in the general population. The cohort design of this study also provided crucial data on the long-term cardioprotective effects of ED. Notably, the risk was reduced most prominently within the first three years of starting treatment, especially for HF (RR 0.94) and MI (RR 0.74). This further supports the use of ED as an early marker for underlying cardiovascular disease, and active treatment of ED could improve overall cardiovascular health.

Other vulnerable populations taking PDE-5 inhibitors for ED, such as patients with rheumatoid arthritis, also have a decreased risk of heart failure, myocardial infarction, and death^14^.

Finally, a retrospective observational study by Kloner et al.^15^ examined the effects of PDE-5 inhibitors on Major Adverse Cardiovascular Events (MACE) and mortality and found a reduced incidence of both. Specifically, there was a 13% reduction in the incidence of MACE (HR 0.87) and a 25% reduction in the risk of overall mortality (HR 0.75). More importantly, they found a 39% reduction in cardiovascular mortality. They also suggested a dose–response relationship, such that a greater PDE-5 inhibitor dosage was associated with fewer cardiovascular events.

### Neutral outcomes

While certain contexts suggest positive outcomes of PDE-5 use in patients with CVD, there are studies in which ED treatment failed to produce any significant results concerning key clinical measures. We categorized these results as neutral outcomes.

One such outcome was found in a randomized controlled trial in which sildenafil was examined in patients with HFpEF and pulmonary hypertension. After 12 weeks of starting treatment, no statistically significant difference was found between the sildenafil and placebo groups in mean pulmonary artery pressure (PAP), pulmonary artery wedge pressure (PAWP), or exercise capacity^16^. This contradicts the improvement in exercise capacity found in Andersen et al.’s^10^ study but confirms its other neutral findings. For instance, no significant changes were observed in the resting PAWP or overall cardiac output at rest. Such neutral outcomes indicate that the benefit of sildenafil may be more prominent during physical exertion than at rest.

Nunes et al.^17^ focused on patients co-prescribed both PDE-5 inhibitors and nitrates, which are typically contraindicated because of the risk of severe hypotension. Interestingly, no significant increase in hypotensive or cardiovascular outcomes was detected in patients co-possessing PDE-5 inhibitors and nitrates compared to those on nitrates alone. However, the study does not suggest the safety of co-prescription; rather, it addresses the possibility of such a drug combination in the presence of careful patient selection and physician supervision.

As for tadalafil, a study investigating its cerebrovascular effects in post-stroke patients with ED did not yield any significant results^18^.

### Negative outcomes

The rest of our findings raised concerns about the potential adverse effects of PDE-5 inhibitors in some high-risk populations. Such findings were categorized as negative outcomes.

As previously mentioned, the use of tadalafil not only failed to provide cerebrovascular benefits, but its long-term use also reduced blood flow in stroke-afflicted regions of the brain. This may increase the risk of cerebrovascular events; hence, tadalafil should be prescribed with caution in post-stroke populations^18^.

Moreover, adverse side effects have been reported with the use of sildenafil, including respiratory infections, headaches, and hypotension. These side effects, in addition to the lack of benefit in the key clinical outcomes mentioned earlier, imply that sildenafil poses a greater risk than benefit in patients with HFpEF and pulmonary hypertension^16^.

### Comparison with existing literature

We compared the findings of our systematic review of PDE-5 inhibitors and cardiovascular outcomes with those of existing systematic reviews and meta-analyses on the topic. For instance, we examined two meta-analyses: one on chronic PDE-5 inhibition^12^ and the other on sildenafil for congenital heart disease-induced pulmonary hypertension^19^. In addition to two systematic reviews/meta-analyses on the long-term cardiovascular effects of PDE-5 inhibitors^20^ and the cardiovascular effects of PDE-5 inhibitors in diabetic populations^21^. The following section discusses the similarities, differences, and new insights of each study.

### Cardioprotective effects

Similar to our review, Gianetta et al.^12^ and Soulaidopoulos et al.^20^ showed the benefits of PDE-5 inhibitors in high-risk patients with CVD. For example, patients with congenital heart disease and pulmonary hypertension taking PDE-5 inhibitors had significant improvement in cardiac geometry and a subsequent improvement in ejection fraction^12^. These findings are consistent with those of the studies in our review, which reported improved exercise-induced cardiac output in post-MI patients^10^. This highlights the potential of PDE-5 inhibitors to produce cardioprotective effects, especially in vulnerable populations.

Andersen et al.^10^ further reinforces the broader cardiovascular benefits of PDE-5 inhibitors. Their findings of a 22% reduction in MACE and a 30% reduction in all-cause mortality align well with the findings of Kloner et al.^15^ in our review, who had similar percentage reductions in both variables.

A more nuanced view was presented by Seidu et al.^21^. When evaluating PDE-5 inhibitor use in patients with diabetes, no significant reduction in MACE or mortality risk compared to non-users was observed. This indicates that PDE-5 inhibitors are less effective in offering cardioprotective effects in patients with diabetes mellitus. This could be attributed to the complexity of diabetes in terms of endothelial function and vascular complications compared with other cardiovascular diseases.

### Hemodynamic effects

In terms of hemodynamic effects, chronic PDE-5 inhibition did not significantly affect systemic blood pressure or systemic vascular resistance index (SVRi)^12^. This translates well with the neutral findings reported in some studies from our review, particularly in patients with HFpEF and pulmonary hypertension and post-MI patients with diastolic dysfunction, where sildenafil showed no significant reduction in pulmonary artery pressure (PAP) or pulmonary artery wedge pressure (PAWP)^10,16^.

In contrast, sildenafil use in the pediatric population with congenital heart disease-induced pulmonary hypertension (CHD-PAH) was efficient in reducing pulmonary pressures, including the mean pulmonary arterial pressure (mPAP) and systolic pulmonary arterial pressure (sPAP)^19^. Although the pediatric population is not the focus of our review, these findings emphasize the notable hemodynamic improvements of PDE-5 inhibitors, particularly in the pulmonary circulation.

Additionally, the findings of Soulaidopoulos et al.^20^ support the notion that the cardiovascular benefits of PDE-5 inhibitors lie in their effects on endothelial function, inflammation reduction, and vascular resistance rather than broad systemic hemodynamic changes. This aligns well with the reduction in inflammation observed in a clinical trial^9^ and the improvements observed in endothelial function and exercise-induced cardiac output in the SIDAMI trial^10^. This highlights the selective hemodynamic benefits of PDE-5 inhibitors under specific conditions.

### Safety and tolerability

The safety profile of PDE-5 inhibitors is consistently described as favorable in the literature, as well as in our review^12,19–21^. The existing literature has emphasized that PDE-5 inhibitors do not increase the incidence of serious adverse events and are generally well tolerated, even in high-risk populations^12,20^.

Although PDE-5 inhibitors did not significantly reduce cardiovascular outcomes in patients with diabetes, their safety profiles were acceptable^21^. This finding is crucial for patients with diabetes, who often have complex medical needs and a higher risk of adverse drug interactions.

However, caution is advised when prescribing PDE-5 inhibitors to certain populations. Particularly patients who had a cerebrovascular stroke^18^, and those taking nitrates^17^.

### Interpretation of findings

The findings of this systematic review suggest that PDE5 inhibitors may provide cardioprotective benefits in select populations, beyond their role in the treatment of erectile dysfunction. The strongest evidence, supported by high-quality cohort studies and one randomized controlled trial, points to a reduction in major adverse cardiovascular events (MACE) and all-cause mortality, particularly in men with existing cardiovascular disease or risk factors. These conclusions were based on moderate-certainty evidence from the GRADE assessment.

Conversely, findings regarding myocardial infarction, heart failure, stroke outcomes, and hemodynamic measures are supported by lower-certainty evidence, often due to smaller sample sizes, inconsistent outcome definitions, and study design limitations. As such, conclusions regarding these endpoints should be interpreted cautiously and considered hypotheses for future research.

### Mechanistic insights and differential effects

The potential mechanisms underlying the cardiovascular benefits of PDE5 inhibitors include improved endothelial function, reduced systemic inflammation, increased nitric oxide availability, and enhanced exercise tolerance. These mechanisms may partly explain the differential effects observed across the various cardiovascular subgroups. For example, the anti-inflammatory properties of tadalafil may be particularly beneficial in hypertensive patients, whereas the limited efficacy of sildenafil in HFpEF and post-stroke populations may reflect different pathophysiological constraints, such as impaired autonomic regulation or compromised cerebral vasculature.

### Limitations of the evidence base

This study has several limitations. First, heterogeneity in study design, intervention type, follow-up duration, and outcome definitions hindered direct comparison and formal meta-analysis. Second, publication bias may be present, particularly as most of the included studies reported positive outcomes, and funnel plots could not be assessed due to the limited number of comparable studies. Third, potential conflicts of interest and industry funding were not consistently reported across studies, raising concerns about selective outcome reporting.

Additionally, the absence of data on female populations limits the generalizability of the findings, and the reliance on observational data in several studies introduces the risk of residual confounding despite methodological adjustments.

### Clinical implications and recommendations

The overall findings support the consideration of PDE5 inhibitors as adjuncts for cardiovascular risk reduction in carefully selected male patients with ED and coexisting cardiovascular risk factors. For patients with stable coronary artery disease, heart failure with reduced ejection fraction, or systemic hypertension, PDE5 inhibitors may provide additional vascular and inflammatory benefits. However, caution is warranted in populations with a history of stroke, advanced heart failure, or concomitant nitrate use, in which the potential risks may outweigh the benefits.

Clinicians are advised to individualize treatment decisions based on cardiovascular risk profiles, concurrent medications, and tolerability. Further high-quality randomized trials are needed to clarify the role of PDE5 inhibitors across a broader spectrum of cardiovascular conditions and to assess their utility in female and elderly populations.

### Future research

Future studies should focus on determining the long-term outcomes of PDE-5 inhibitors in high-risk groups, such as patients with HFpEF and pulmonary hypertension, or individuals recovering from a stroke with compromised cerebral blood flow. To thoroughly evaluate the safety and effectiveness of these treatments across different populations, larger multicenter trials with prolonged follow-up are essential. Moreover, further research into the potential anti-inflammatory properties of tadalafil in lowering cardiovascular risk would offer valuable insights for clinicians managing patients with both ED and cardiovascular disease^9^. Furthermore, prospective investigations on the combined use of PDE-5 inhibitors and nitrates would be instrumental in corroborating the results of Nunes et al.^17^ and guiding potential updates to clinical practice guidelines. Finally, future studies should consider shifting the focus to the female population to broaden the understanding of the effects of PDE-5 inhibitors on cardiovascular outcomes in both sexes.

## Conclusion

This systematic review highlights the dual benefits of PDE5 inhibitors in treating ED and improving cardiovascular outcomes in patients with coexisting CVD. These medications show promise in reducing major adverse cardiovascular events (MACE), myocardial infarction, heart failure, and overall mortality, particularly with long-term use. Improvements in endothelial function, systemic inflammation, and exercise capacity underscore the mechanisms underlying these benefits. However, the variability in outcomes across different populations, ranging from cardioprotective effects in high-risk groups to adverse effects in stroke survivors, indicates the need for individualized treatment strategies.

Clinicians should consider the broader cardiovascular implications of PDE5 inhibitors and tailor treatment based on patient-specific factors, such as concurrent conditions and medication regimens. Future research focusing on long-term outcomes in diverse populations, including females, and exploring combined therapies, will further elucidate the potential of PDE5 inhibitors in cardiovascular risk management.
